# Recycling mushroom residue as a soil amendment: pH-associated attenuation of blueberry crown gall severity diverging from enhanced rhizosphere denitrification

**DOI:** 10.3389/fmicb.2026.1952468

**Published:** 2026-09-15

**Authors:** Liu Hong, Zhang Tianlun

**Affiliations:** 1 Department of Chemical Engineering, Fushun Vocational and Technical College, Fushun, Liaoning, China; 2 School of Economics and Management, Tsinghua University, Beijing, China

**Keywords:** blueberry, crown gall disease, *nirK*, *nirS*, rhizosphere microenvironment, soil amendment

## Abstract

Cold region blueberry production faces constraints from unsuitable soils and crown gall disease caused by Agrobacterium tumefaciens. To investigate whether amendment-mediated soil changes regulate this disease, a 3 × 3 factorial pot experiment was conducted with three amendments [CK (none), T1 (peat + sulfur), and T2 (peat + mushroom residue + sulfur)] under three water regimes, using artificial stem-wound inoculation with A. tumefaciens. We quantified rhizosphere properties, denitrification genes (*nirS/nirK*), the pathogenicity marker *virD2*, potential denitrification rate (Dr), and crown gall severity assessed on a 0–9 scale at 30 days post-inoculation. T2 resulted in the lowest pH (4.99) and the highest nutrient pools. *nirS* abundance followed T2 > T1 > CK, whereas *nirK* showed no significant response. Dr followed T1 > T2 > CK (1.66–31.33 mg⋅kg^−1^⋅h^−1^). *virD2* and gall severity decreased in the order CK > T1 > T2. Although disease incidence reached 100% at the treatment-combination level, and the disease index declined from 65.4 (CK) to 23.5 (T2). The severity pattern aligned with pH and *virD2* but diverged from Dr, indicating that T2 attenuated symptoms primarily through pH-associated reduction in *virD2* abundance rather than through enhanced denitrification potential. The contrasting response patterns between enhanced denitrification (T1) and reduced severity (T2) further imply that these two effects were not directly linked but may represent independent responses to the amendments.

## 1 Introduction

Blueberries are of considerable horticultural and economic value, and in recent years the cultivated area in the cold regions of north-eastern China has continued to expand. However, blueberry production in these regions faces constraints such as unsuitable soil pH, poor aeration and drainage, low organic matter content, and nutrient imbalances (Lee S. I. et al., 2021); these factors not only inhibit plant growth but may also alter rhizosphere microbial metabolism and exacerbate soil-borne diseases. Soil improvement can enhance the physicochemical environment of the root zone, while also influencing nitrogen cycling and disease incidence by reshaping functional microbial communities in the rhizosphere (Li et al., 2024).

The “Northland” variety (Vaccinium corymbosum × V. angustifolium “Northland”) is a typical semi-dwarf blueberry. It is highly cold-tolerant and maintains a stable, high yield, which is why it is widely cultivated in the cold regions of north-eastern China. Crown gall disease, caused by the soil-borne pathogen Agrobacterium tumefaciens, invades the plant through wounds and induces the formation of galls (crown galls), posing a significant threat to blueberry production. By using “Northland” as a model system, it is possible to analyse the relationship between soil improvement, functional rhizosphere microorganisms, and crown gall disease, thereby providing a basis for soil management and ecological control in blueberry production in cold regions. Existing studies on blueberry soil improvement have mainly focused on acidification regulation, organic matter supplementation, and aeration/drainage enhancement. Blueberry has shallow fine roots that are highly sensitive to soil pH, moisture, and nitrogen form; these factors often become major constraints to growth and yield (He et al., 2023). Soil pH not only determines nutrient availability but also acts as a key environmental driver of microbial community structure (Ma et al., 2025). Furthermore, amendment materials such as peat, sulfur, and mushroom residue can optimize soil chemical properties. They also alter carbon supply, oxygen diffusion, water retention, and nitrogen transformation processes. These changes, in turn, can significantly restructure rhizosphere functional microbial communities (Kang et al., 2026).

Recently, two studies on blueberry crown gall were published, one reported the complete genome sequence and comparative genomic analysis of the pathogen Agrobacterium fabrum (Wang A. et al., 2024), and the other explored the use of bacteriophages for biological control of stem crown gall in highbush blueberry (Chen et al., 2024).

This study intends to test three hypotheses. First, (H1) compared with the T1 treatment (peat + sulfur), the T2 treatment (peat + mushroom residue + sulfur) would show stronger effects in both reducing rhizosphere pH and enriching nutrient pools. Second, (H2) both *virD2* abundance and crown gall disease severity exhibited a decreasing trend along the order of CK, T1, and T2. Third, (H3) the potential denitrification rate (Dr) showed no direct association with disease severity, which suggests the existence of an independent regulatory pathway (Che et al., 2023). In this study, the effects of three soil amendments (CK, T1, T2) and three irrigation regimes on the rhizosphere physicochemical properties, denitrification gene abundances (*nirS/nirK*), potential denitrification rate (Dr), pathogenic marker gene *virD2*, and crown gall phenotype of “Northland” blueberry were quantified separately (Phan and Sabaratnam, 2012; Lee S.-Y. et al., 2021).

## 2 Materials and methods

### 2.1 Test specimens

The blueberry variety used in the experiment was 1-year-old tissue-cultured seedlings of “Northland.” Before transplanting, uniformly growing plants were selected, graded, and then randomly assigned to treatment groups. The test soil was collected from the plow layer (0–20 cm) of a blueberry orchard in Jingyu County, Jilin Province; after sampling, impurities were removed, the soil was thoroughly mixed, and passed through a 2 mm sieve for later use. Artificial inoculation with Agrobacterium tumefaciens strain BNCC180854 (BeNa Culture Collection Center, China) was performed to induce crown gall disease, and the amendment materials, including peat, sulfur powder, and mushroom residue, were all air-dried and sieved before use. Plastic pots with top diameter 25 cm and height 20 cm were used. Each pot contained 2.5 kg of air-dried soil. River sand or perlite was mixed at the same proportion (10%, v/v) to ensure uniform baseline aeration.

### 2.2 Experimental design

The experiment was arranged in a completely randomized design with 27 pots in total (3 amendments × 3 water regimes × 3 replicate pots). Each pot represented an independent experimental unit and contained one blueberry plant. For each treatment combination, the three replicate pots were randomly assigned to one of three purposes: destructive sampling for rhizosphere soil collection, disease assessment, or backup. All pots were independently randomized at the time of treatment allocation; thus, the three replicate pots per treatment combination constituted independent biological replicates rather than subsamples of a common replicate. Prior to transplanting, uniformly growing plants were randomly assigned to pots to minimize initial variation. For each measurement set, the individual pot, each being an independently randomized experimental unit, was treated as the statistical unit (*n* = 3 per treatment combination), whereas qPCR technical replicates were averaged and were not treated as independent observations.

The treatments were as follows:

CK (control): no amendment added.

T1: peat (10%, v/v) + sulfur powder (1.5 kg⋅m^−3^).

T2: peat (10%, v/v) + mushroom residue (10%, v/v) + sulfur powder (1.5 kg⋅m^−3^).

The application ratios were determined as follows, with each component selected based on distinct rationales:

Peat at 10% (v/v):This is a standard substrate amendment ratio widely used in blueberry cultivation to improve soil physical structure, water-holding capacity, and organic matter content (Lee S. I. et al., 2021).

Sulfur powder at 1.5 kg⋅m^−3^:This rate follows the locally recommended guideline for acidifying soils to the target pH range of 4.5–5.5, which is optimal for blueberry growth and has been validated in previous regional studies (He et al., 2023).

Mushroom residue at 10% (v/v):This rate was selected based on preliminary dose–response trials (conducted over 60 days prior to this experiment), in which 10% (v/v) provided the greatest increase in soil organic matter without causing salt stress (electrical conductivity < 1.5 mS⋅cm^−1^) or growth inhibition in blueberry seedlings. Lower rates (5%) showed limited organic matter enrichment, while higher rates (15% and 20%) led to excessive soluble salt accumulation and reduced plant height.

The combined use of these three materials in T2 was intended to achieve synergistic effects on soil acidification, nutrient enrichment, and organic matter supplementation.

All amendment materials were thoroughly mixed with the test soil before potting. After filling the pots, the soil was equilibrated for 15 days, then blueberry plantlets were transplanted. The experiment was conducted under greenhouse conditions. All treatments received consistent basic water and fertilizer management.

### 2.3 Water treatment

Three water regimes were set:

W1 (moderate water): soil moisture at 60%–70% of field water holding capacity.

W2 (high water): soil moisture at 80%–100% of field water holding capacity.

W3 (low water): soil moisture at 30%–40% of field water holding capacity.

Field water holding capacity (θWHC, gravimetric water content, %) was measured for each potted substrate. Daily soil moisture was adjusted by the weighing method. Dry soil weight (W_dry) was determined for each pot. The target total weight for each treatment was calculated as Wtarget = Wdry × (1 + θtarget), where θtarget is the corresponding absolute water content. Pots were weighed at fixed times daily. If the weight dropped below the lower limit, water was added to reach the upper limit. If it exceeded the upper limit, no water was added. Sampling and measurements were conducted after the target moisture gradients were stably maintained for 14 days.

### 2.4 Sample collection

At sampling, blueberry plants were removed intact from pots. Loosely attached bulk soil was gently shaken off. Rhizosphere soil was collected by scraping the 0–5 mm layer from root surfaces using the root-shaking method.

For each amendment treatment under each water regime, three biological replicates were sampled, yielding 27 rhizosphere soil samples (3 amendments × 3 water regimes × 3 replicates). Each sample was passed through a 2 mm sieve to remove roots and gravel and divided into two portions. One portion was air-dried for physicochemical analyses, and the other was stored at −80°C for DNA extraction and qPCR.

### 2.5 Inoculation with *agrobacterium tumefaciens* and disease assessment

Artificial inoculation was performed within 3 days after blueberry transplanting. *Agrobacterium tumefaciens* strain BNCC180854 was cultured in YEB liquid medium at 28 °C and 180 r⋅min^−1^ with shaking until the logarithmic growth phase (OD600 = 0.8–1.0). According to the supplier’s technical data for this strain (BeNa Culture Collection, China), an OD600 of 1.0 under the recommended culture conditions corresponds to approximately 1.0 × 108 CFU⋅mL^−1^. Three to five needle pricks (approximately 1–2 mm deep) were made at the base of each blueberry stem using a sterile needle. A total volume of 100 μL of the bacterial suspension was evenly dropped onto the wounded sites of each plant (i.e., approximately 20–33 μL per wound, depending on the exact number of pricks). All treatments (CK, T1, and T2) received exactly the same inoculum concentration (approximately 1.0 × 108 CFU⋅mL^−1^), total inoculation volume (100 μL per plant), and wounding procedure (3–5 needle pricks per stem base) to ensure uniformity across experimental groups. After inoculation, plants were observed regularly for symptoms. Disease assessment was conducted at 30 days post-inoculation. Rhizosphere soil samples collected from the destructive-sampling pots (as described in Section “2.4 Sample collection”) were used for DNA extraction, and the *virD2* gene copy number was quantified by qPCR as a proxy for the abundance of A. tumefaciens in the rhizosphere, although this measurement reflects target-gene abundance rather than viable cell counts or gene expression activity. Disease severity was assessed at 30 days post-inoculation using a 0–9 rating scale adapted from standard crown gall scoring protocols: 0 = no symptoms; 1 = slight callus proliferation ( < 1 mm) at the inoculation site; 3 = distinct small galls (1–3 mm diameter, 1–2 per plant); 5 = moderate galls (3–7 mm, 2–4 per plant, partially extending to crown); 7 = large galls (7–15 mm, multiple, extending around the root collar); 9 = severe multiple large galls ( > 15 mm) with obvious plant growth suppression. Disease incidence was calculated as the percentage of symptomatic plants per treatment group. Disease index (DI) was calculated as DI = [Σ(disease grade × number of plants at that grade)/(total plants × 9)] × 100. Gall number per plant, maximum gall diameter (measured with digital calipers), and gall fresh weight were recorded. Gall dry weight was determined after oven-drying at 60 °C to constant weight (dry/fresh weight ratio ≈ 0.18) (Hildebrand et al., 2001).

### 2.6 Determination of soil physicochemical properties

Soil physicochemical properties were determined according to the methods described in Methods of Soil Agricultural Chemical Analysis. The specific procedures were as follows (Sparks et al., 1996):

pH: measured with a pH meter at a soil-to-water ratio of 2.5:1, with 30 min extraction.

Soil moisture (SM): determined by the oven-drying method.

Soil organic matter (OM): determined by the potassium dichromate external heating method.

Total nitrogen (TN): determined by the Kjeldahl method.

Total phosphorus (TP): determined by NaOH fusion followed by Mo-Sb colorimetry.

Ammonium nitrogen (NH_4_^+^-N): determined by KCl extraction followed by indophenol blue colorimetry.

Nitrate nitrogen (NO_3_^–^-N): determined by ultraviolet spectrophotometry.

Available phosphorus (AP): determined by NaHCO3 extraction followed by Mo-Sb colorimetry.

### 2.7 Soil DNA extraction and real-time quantitative PCR (qPCR)

Total soil DNA was extracted using a commercial kit according to the manufacturer’s instructions. DNA quality and concentration were checked by 1% agarose gel electrophoresis and NanoDrop 2000 spectrophotometer, respectively. Qualified samples were used for qPCR analysis. The abundances of *nirS* (primers cd3aF/R3cd; Throbãck et al., 2004)), *nirK* (primers FlaCu/R3Cu; Braker et al., 1998)), and the pathogenicity gene *virD2* of Agrobacterium tumefaciens were quantified by qPCR using primers *virD2*-F (5′-ATGCCCGATCGAGCTCAAGT-3′) and *virD2*-R (5′-TCGTCTGGCTGACTTTTCGTCATAA-3′), yielding a 224 bp product (Haas et al., 1995). These primers have been validated for specific amplification of virD2 across multiple Agrobacterium strains in previous studies, and amplification specificity in our samples was further confirmed by melt curve analysis showing a single peak. Reactions were performed on a LightCycler^®^ 480 II (Roche, Switzerland) using SYBR^®^ Green Premix Ex Taq™ (Takara, Japan). The amplification program was: 95 °C for 30 s pre-denaturation; 40 cycles of 95 °C for 5 s denaturation, annealing at 57 °C for nirS/nirK or 60 °C for *virD2* for 30 s, and extension at 72 °C for 30 s; followed by melting curve analysis (65–95 °C, 0.5 °C⋅s^−1^) to verify amplification specificity.

Standard curves were generated using 10-fold serial dilutions of recombinant plasmids containing the target fragments. The amplification efficiency ranged from 85 to 95%, with R^2^ > 0.99. Three technical replicates were performed per sample. Gene copy numbers were expressed as copies per gram of dry soil (copies⋅g^−1^ dry soil).

### 2.8 Determination of potential denitrification rate

The potential denitrification rate was determined using the nitrate residual method (Zhou et al., 2018). Fresh soil equivalent to 1 g oven-dry soil was placed in a 50 mL Erlenmeyer flask. Then 20 mL of 10 g⋅L^−1^ KNO_3_ solution was added. The flask was stoppered and sealed with Parafilm to create microaerobic to anaerobic conditions; no additional gas flushing was applied. Blank controls (KNO_3_ solution without soil) and sterilized soil controls (autoclaved at 121 °C for 20 min) were included to account for abiotic NO_3_^–^ consumption. It was incubated at 35 °C for 3 days. Although this incubation time is longer than in some previous studies, we adopted it to ensure detectable NO_3_^–^ consumption across all treatments, including those with low denitrification activity. Preliminary tests confirmed that NO_3_^–^ consumption was linear over the first 72 h under our conditions, and final NO_3_^–^ concentrations remained above 50% of initial values in all treatments, indicating that substrate depletion was not a major constraint. After incubation, the mixture was centrifuged at 3500 r⋅min^−1^ for 10 min. The supernatant was filtered, and the NO_3_^–^-N content in the filtrate was measured.

To determine the initial NO_3_^–^ content, another portion of fresh soil of the same weight was extracted with 20 mL of 4 mol⋅L^−1^ KCl solution. After centrifugation and filtration, the NO_3_^–^-N content was measured. The externally added NO_3_^–^-N was added to obtain the initial total amount. The NO_3_^–^-N consumption rate was calculated as:


$$\mathrm{Dr}=[\left({\mathrm{NO}}_{3}^{-}-N\_\mathrm{initial}-{\mathrm{NO}}_{3}^{-}-N\_\mathrm{final}\right)/$$

(soildryweight×incubationtime)]

where NO_3_^–^-N is expressed in mg, soil dry weight in kg, and incubation time in hours. The final rate is expressed as mg⋅kg^−1^⋅h^−1^. Because this method measures total NO_3_^–^ removal without distinguishing denitrification from dissimilatory nitrate reduction to ammonium (DNRA) or nitrate assimilation, we refer to this parameter as the nitrate-removal-based potential denitrification rate (Dr).

However, this method assumes that all NO_3_^–^ loss is due to denitrification. In acidic carbon-rich soils such as T2, dissimilatory nitrate reduction to ammonium (DNRA) may also consume NO_3_^–^, potentially leading to an underestimation of the actual denitrification rate in those treatments.

### 2.9 Data analysis

All statistical analyses were performed using SPSS 26.0 and R 4.3.1.

qPCR copy numbers were log10-transformed prior to analysis to improve variance homogeneity and normality.

ANOVA was used to test the effects of soil amendment (CK, T1, T2), water treatment (W1, W2, W3), and their interaction on soil physicochemical properties, gene abundances, nitrate-removal-based potential denitrification rate, and disease indicators. The significance level was set at α = 0.05. Differences among treatments were compared using Duncan’s multiple range test. Effect size (partial η^2^) was calculated to evaluate the practical significance of treatments on denitrification rate and disease indicators. For disease phenotypes, two-way ANOVA was used to test the main effects of amendment and water regime and their interaction on disease index, gall number, gall diameter, and gall fresh weight. We acknowledge that disease incidence (binomial) and severity scores (ordinal) are more appropriately analyzed using generalized linear models or nonparametric methods; however, given the small sample size (*n* = 3 per treatment combination) and the exploratory nature of this study, we used ANOVA as a conservative approach to detect overall treatment effects. For denitrification functional genes and potential denitrification rate, one-way ANOVA was used with the nine treatment combinations as the single factor. This approach was chosen because the limited sample size (*n* = 3 per treatment combination) precluded meaningful two-way ANOVA for these variables, as partitioning the data into main effects and an interaction would have reduced the effective sample size per cell and substantially increased the risk of Type II errors. When significant differences were detected, Duncan’s multiple range test was used for *post-hoc* comparisons. Normality and homogeneity of variances were verified prior to all ANOVAs using the Shapiro-Wilk test and Levene’s test, respectively.

Spearman correlation analysis was performed to assess pairwise relationships among *nirS/nirK* abundances, potential denitrification rate, soil properties, and disease indicators. Separate correlation analyses were conducted for each water treatment to identify changing patterns of driving factors under different moisture conditions. For multiple comparisons in correlation analyses, *P*-values were adjusted using the Benjamini-Hochberg false discovery rate (FDR) procedure; only correlations with *q* < 0.05 were considered significant.

Correlation heatmaps were generated using the pheatmap package in R.

Redundancy analysis (RDA) and random forest analysis were performed using the vegan and randomForest packages in R, respectively, to identify key environmental predictors of denitrification function and disease indicators. For RDA, explanatory variables were selected using forward selection with 999 Monte Carlo permutation tests; only variables with significant independent effects (*P* < 0.05) were retained in the final model. The significance of the overall RDA model and each constrained axis was assessed by permutation tests. To assess the robustness of the RDA results, two sensitivity analyses were conducted: (1) leave-one-out RDA, in which each of the 27 samples was sequentially omitted and the model was refit to examine the stability of the adjusted R^2^; and (2) partial RDA conditioned on sampling period (A, B, C) to account for potential temporal variation in environmental conditions. Random forest was run with 200 iterations (ntree = 500); variable importance was measured as %IncMSE. The consistency between random forest and RDA results was used as a cross-validation check. Given the limited sample size (*n* = 27) and multiple predictors, these results are exploratory and correlational, not causal.

Multiple stepwise regression analysis (SPSS 26.0 stepwise module) was used to identify key predictors of denitrification rate. The potential denitrification rate was set as the dependent variable, and *nirS* abundance, *nirK* abundance, and major physicochemical factors (pH, OM, SM, NH_4_^+^-N, NO_3_^–^-N, etc.) were included as independent variables.

For the response fingerprint, the standardized mean difference (SMD) between each amendment treatment and its paired control (CK) within the same water period was computed on log10-transformed abundances. Because the log10 transformation requires values strictly above zero, abundances at or below the reliable quantification limit of the qPCR assay were recorded as zero in this computation; when fewer than two of the three replicate abundances within a group had quantifiable values, the within-group log10 variance was undefined, the SMD could not be estimated, and the cell was marked as non-estimable (’x’).

## 3 Results

### 3.1 Analysis of soil physicochemical properties

Soil physicochemical properties responded differently to amendment and water treatments. Water regimes primarily affected soil moisture and mineral nitrogen, whereas amendment treatments exerted stronger effects on soil pH and total nutrient pools (Figure 1).

**FIGURE 1:**
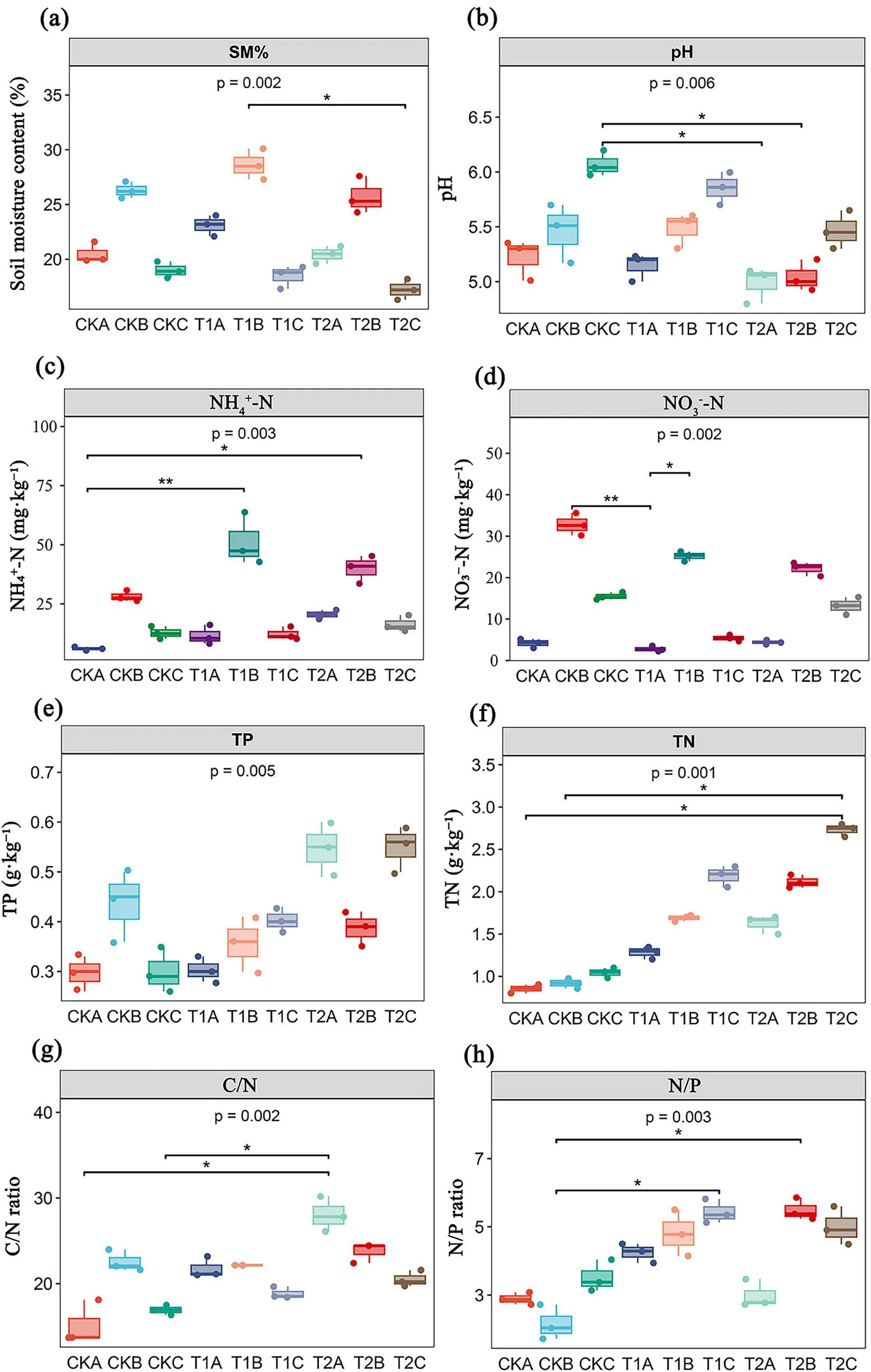
Variations of soil physicochemical characteristics across different amendment treatments and sampling periods.**(a)** Soil moisture (SM); **(b)** pH; **(c)** NH_4_^+^-N; **(d)** NO_3_^–^-N; **(e)** total phosphorus (TP); **(f)** total nitrogen (TN); **(g)** C/N ratio; **(h)** N/P ratio. CK means the control; T1 and T2 are two soil-improved treatments; A, B, C correspond to suitable-water (W1), high-water (W2) and low-water (W3) periods, respectively. Values are means ± SD (*n* = 3). Asterisk denotes significant difference at (*p* < 0.05 level).

Soil moisture (SM) was mainly governed by water regimes (high water > moderate water > low water), with limited regulation by amendments; only T2 showed slightly lower water retention under the low-water condition (W3).

Soil pH varied with both amendment and water regimes. T2 consistently produced lower pH than CK and T1 across all moisture levels, with the lowest value (4.99) observed under moderate water (W1). Inorganic nitrogen (NH_4_^–^-N and NO_3_^–^-N) was generally higher under high water (W2) than under moderate (W1) and low water (W3), although the magnitude of this response varied among amendment treatments.

For total nutrients, T2 showed the greatest enrichment of total nitrogen (TN) across all moisture levels, whereas the treatment ranking for total phosphorus (TP) varied among water regimes. Under low water (W3), TN in T2 (2.73 g⋅kg^−1^) was approximately 2.6 times that in CK (1.04 g⋅kg^−1^). The C/N and N/P ratios also differed among amendment–water combinations.

In summary, water regimes mainly regulated soil water supply and short-term pulses of inorganic nitrogen, whereas amendment treatments had more prominent and consistent effects on total nutrient pools and stoichiometric ratios. This differentiation between short-term supply (water-driven) and long-term reservoir regulation (amendment-driven) provides the physicochemical context for interpreting the subsequent responses of denitrification functional genes, potential denitrification rate, and pathogen-related markers.

### 3.2 Responses of functional genes and potential denitrification rate to amendment and water treatments

The response patterns of denitrification functional gene abundances and potential denitrification rate (Dr) to amendment and water treatments differed by gene type (Figure 2). ANOVA showed that *nirS* abundance, *virD2* abundance, and Dr were significantly affected by the experimental treatments (*p* = 0.035, *p* = 0.008, and *p* = 0.005, respectively), whereas *nirK* abundance was insensitive to the treatments (*p* = 0.283).

**FIGURE 2:**
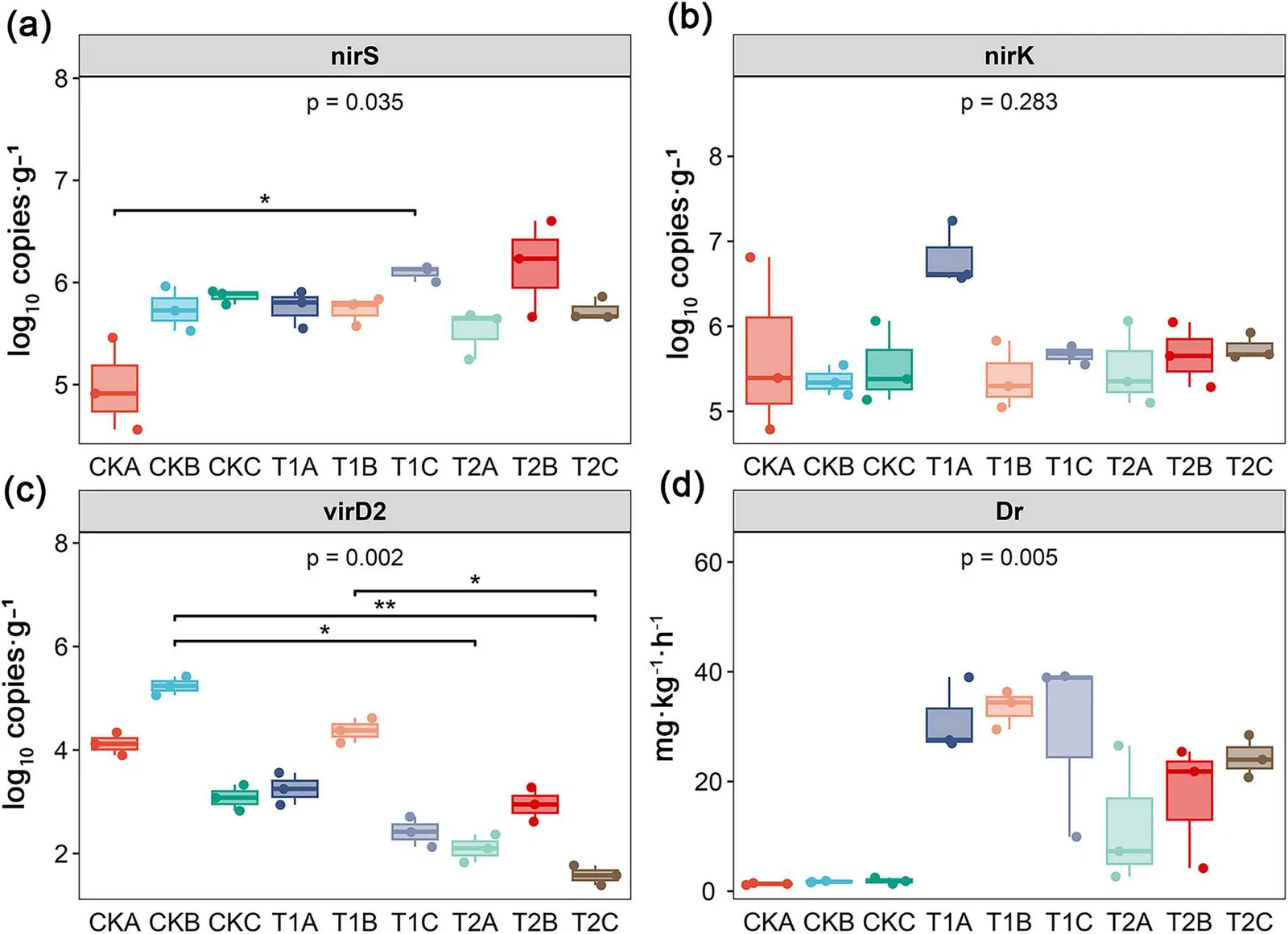
Variations of functional gene abundance and potential denitrification rate across different amendment treatments and sampling periods. **(a)** *nirS* gene abundance; **(b)** *nirK* gene abundance; **(c)** *virD2* gene abundance; **(d)** potential denitrification rate (Dr). CK, control; T1 and T2 are two soil-amended treatments; A, B, C correspond to suitable-water (W1), high-water (W2) and low-water (W3) periods, respectively. The gene copy numbers were log_10_ transformed. Values are means ± SD (*n* = 3). Different letters above bars indicate significant differences among treatments at *p* < 0.05 (Duncan’s test). Significant differences are marked when *p* < 0.05. Text labels CKA, CKB, CKC, T1A, T1B, T1C, T2A, T2B and T2C indicate treatment-period centroids. *,**Indicate statistical significance levels, representing *p* < 0.05 and *p* < 0.01, respectively.

The abundance of *nirS* varied among amendment–water combinations. Its overall abundance was higher in T1 and T2 than in CK. In contrast, *nirK* abundance did not differ significantly among treatments, indicating a weaker response of *nirK*-type denitrifiers to the experimental conditions.

The pathogenicity marker gene *virD2* was detected in all treatments. Its mean abundance decreased from CK (4.15 ± 0.95 log10 copies⋅g^−1^) to T1 (3.35 ± 0.89) and T2 (2.21 ± 0.64), and was generally higher under W2 than under W1 and W3. In contrast, Dr followed the order T1 (31.33 ± 9.41 mg⋅kg^−1^⋅h^−1^) > T2 (17.92 ± 10.22) > CK (1.66 ± 0.40).

In summary, *nirS*, *nirK*, *virD2*, and Dr showed distinct response patterns to amendment and water treatments. Amendment treatments increased denitrification potential, particularly in T1, whereas *virD2* abundance declined progressively from CK to T2. These contrasting response patterns indicate that denitrification potential and pathogen-marker abundance did not vary in a single coordinated direction, with the former peaking in T1 and the latter lowest in T2.

### 3.3 Associations of soil physicochemical factors with denitrification functional genes and disease indicators

Spearman correlation analysis (Figure 3) showed that potential denitrification rate (Dr) was significantly positively correlated with total nitrogen (TN), organic matter (OM), available phosphorus (AP), and the N/P ratio. Among these, TN and N/P had the strongest correlations with Dr, while OM and AP showed moderate positive correlations. Similarly, *nirS* abundance was also significantly positively correlated with TN and N/P.

**FIGURE 3:**
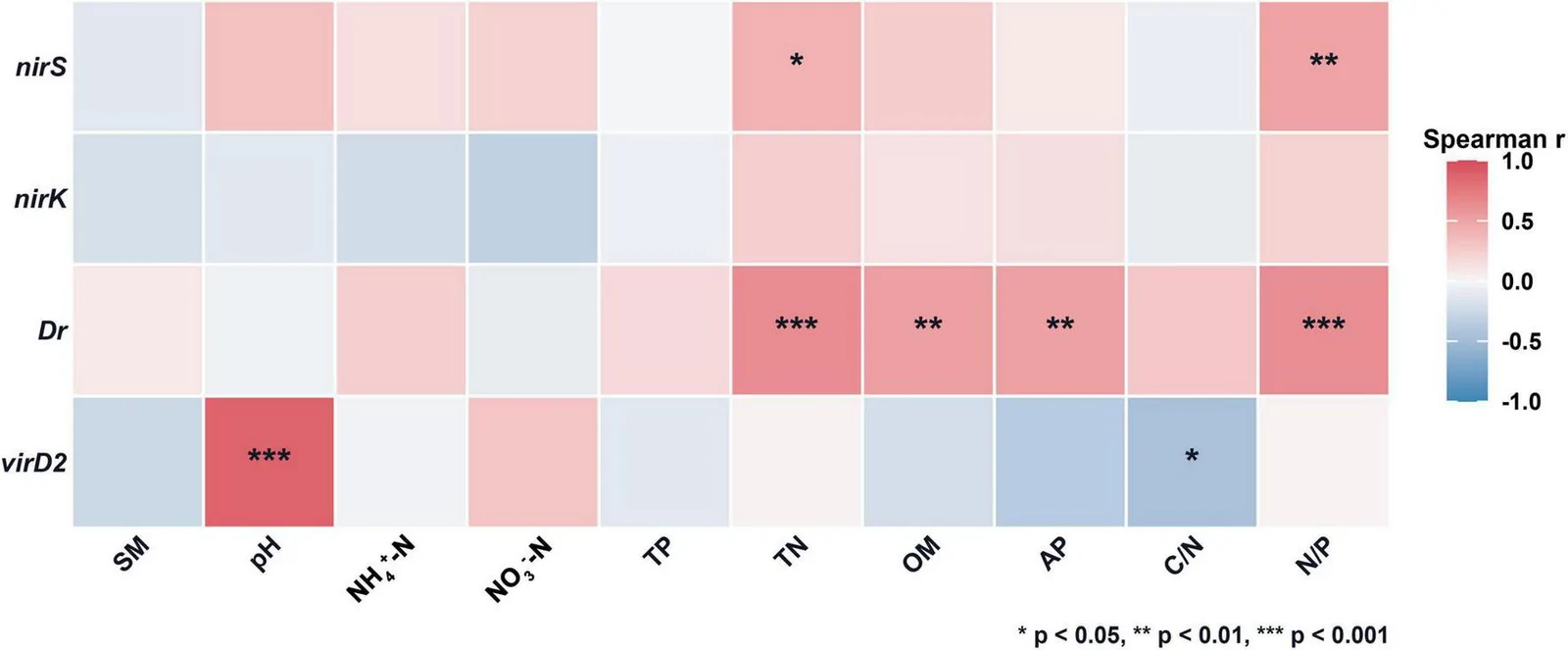
Spearman correlation heatmap linking soil properties with denitrification function and the crown gall disease-related marker. Soil variables are shown on the *x*-axis, and *nirS*, *nirK*, Dr and *virD2* are shown on the *y*-axis. Colors indicate Spearman’s r, with red denoting positive correlations, blue denoting negative correlations and white indicating correlations close to zero. Numerical correlation coefficients are not printed in the cells; asterisks indicate raw Spearman-test significance: *p < 0.05, **p < 0.01 and ***p < 0.001. *nirS, nirK* and Dr were log10-transformed, whereas *virD2* was transformed as log10(virD2 + 1). After Benjamini–Hochberg FDR correction (*q* < 0.05), the following correlations remained significant: pH-*virD2*, TN-Dr, N/P-Dr, OM-Dr, AP-Dr, and N/P-*nirS*; the *virD2*-C/N correlation was significant at the raw *p*-level only.

The pathogenicity marker gene virD2 was significantly positively correlated with soil pH and significantly negatively correlated with the C/N ratio. However, virD2 showed no significant correlations with Dr or with any nitrogen or phosphorus nutrient indices, indicating that the pathogen marker responded to different environmental drivers than those governing denitrification function.

Network analysis (Figure 4) further revealed that Dr was located as a hub node, directly connecting to TN, OM, AP, and N/P. *nirS* was mainly positively correlated with OM, N/P, and C/N, whereas *nirK* abundance was negatively correlated with soil moisture (SM). In contrast, *virD2* maintained only a single edge connection with pH and had no associations with Dr or denitrification functional genes.

**FIGURE 4:**
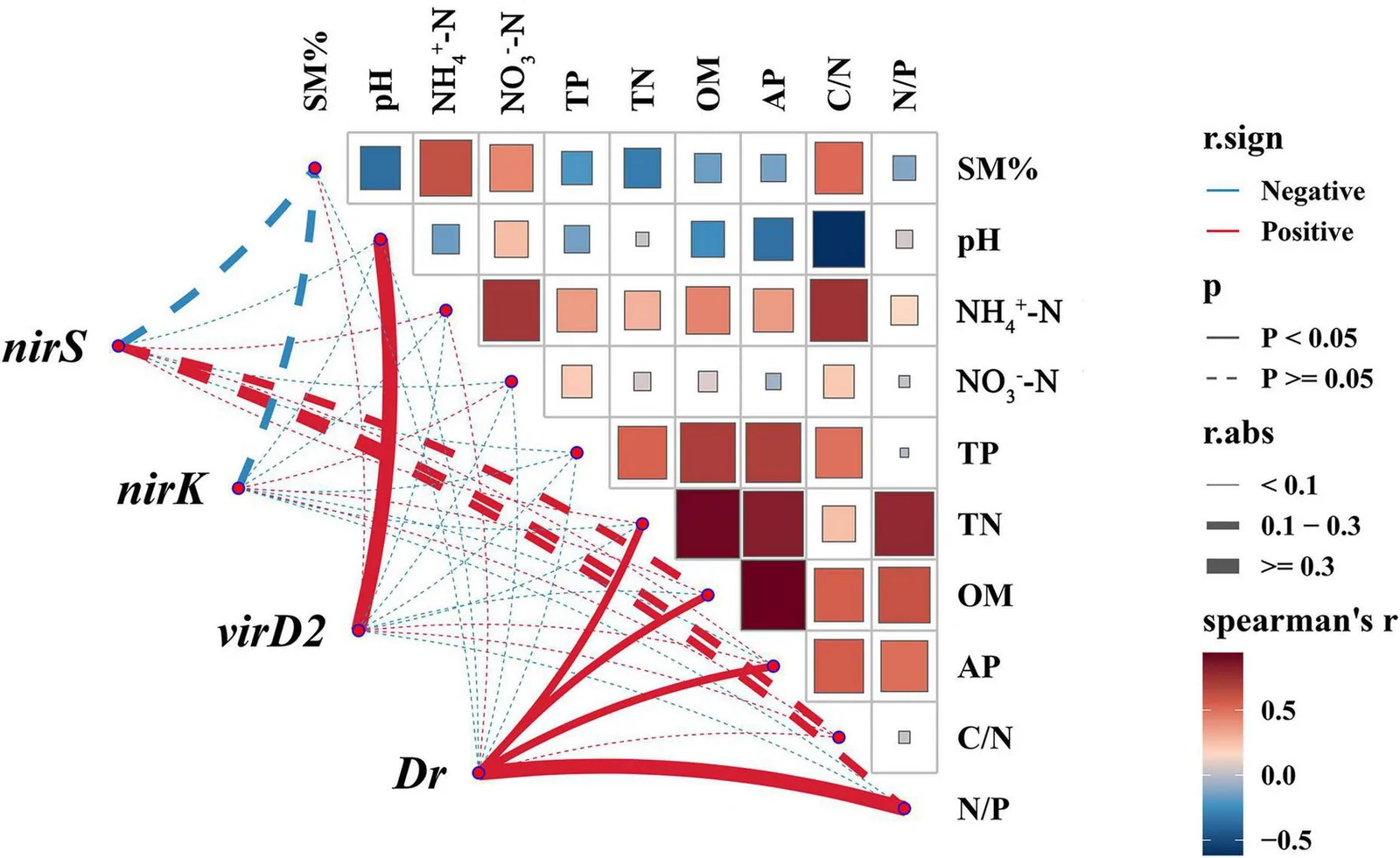
Pairwise correlations among soil physicochemical parameters, functional gene abundance and potential denitrification rate (Dr). The heat-map displays Spearman’s *r* values for soil physicochemical factors. In the network diagram, solid red lines mean significant correlations (*p* < 0.05), dashed blue lines represent non-significant relationships, and line thickness reflects correlation magnitude.

In summary, soil pH showed the strongest association with the crown gall disease-related marker gene *virD2*, whereas TN, N/P, OM, and AP exhibited more consistent associations with Dr. This difference in correlation patterns suggests that the environmental factors driving denitrification potential differ considerably from those related to the pathogen marker gene, with pH playing a unique role in the latter.

### 3.4 Identification of environmental drivers of denitrification function and disease indicators

To remove collinearity among environmental factors and quantify their independent contributions, redundancy analysis (RDA) and random forest analysis were applied. Key conclusions were validated by response fingerprint plots.

In the RDA (Figure 5), RDA1 and RDA2 explained 68.79% and 26.77% of the constrained variance, respectively. These values represent the proportion of explained variance among the constrained axes, not the total variance of the response variables. Samples separated along the RDA1 axis into two groups: CK located in the negative direction, while T1 and T2 concentrated in the positive direction. Dr was positioned in the positive direction of RDA1, *nirS* in the lower-right quadrant, and nirK in the upper-right quadrant, indicating divergent response directions among the three variables. The overall model was significant (*F* = 2.819, *P* = 0.004). The variables retained in the model were pH, NH_4_^–^-N, NO_3_^–^-N, TP, AP, C/N, and N/P. Among these, N/P, NO_3_^–^-N, and NH_4_^–^-N had significant independent effects on the response variables (*P* < 0.05). *To evaluate the robustness of the RDA model, leave-one-out sensitivity analysis was performed by sequentially omitting each of the 27 samples and refitting the model. The adjusted R^2^ values from the 27 refits ranged from 0.284 to 0.396 (median = 0.324), with the original adjusted R^2^ (0.329) closely matching the median, indicating that the model was not unduly influenced by any single sample. Additionally, a partial RDA conditioned on sampling period showed that the soil explanatory matrix remained significant after accounting for temporal variation (Pseudo-F = 2.525, permutation *P* = 0.0088, adjusted partial R^2^ = 0.308), with RDA1 and RDA2 explaining 41.51% and 7.33% of the conditional total variance, respectively.

**FIGURE 5:**
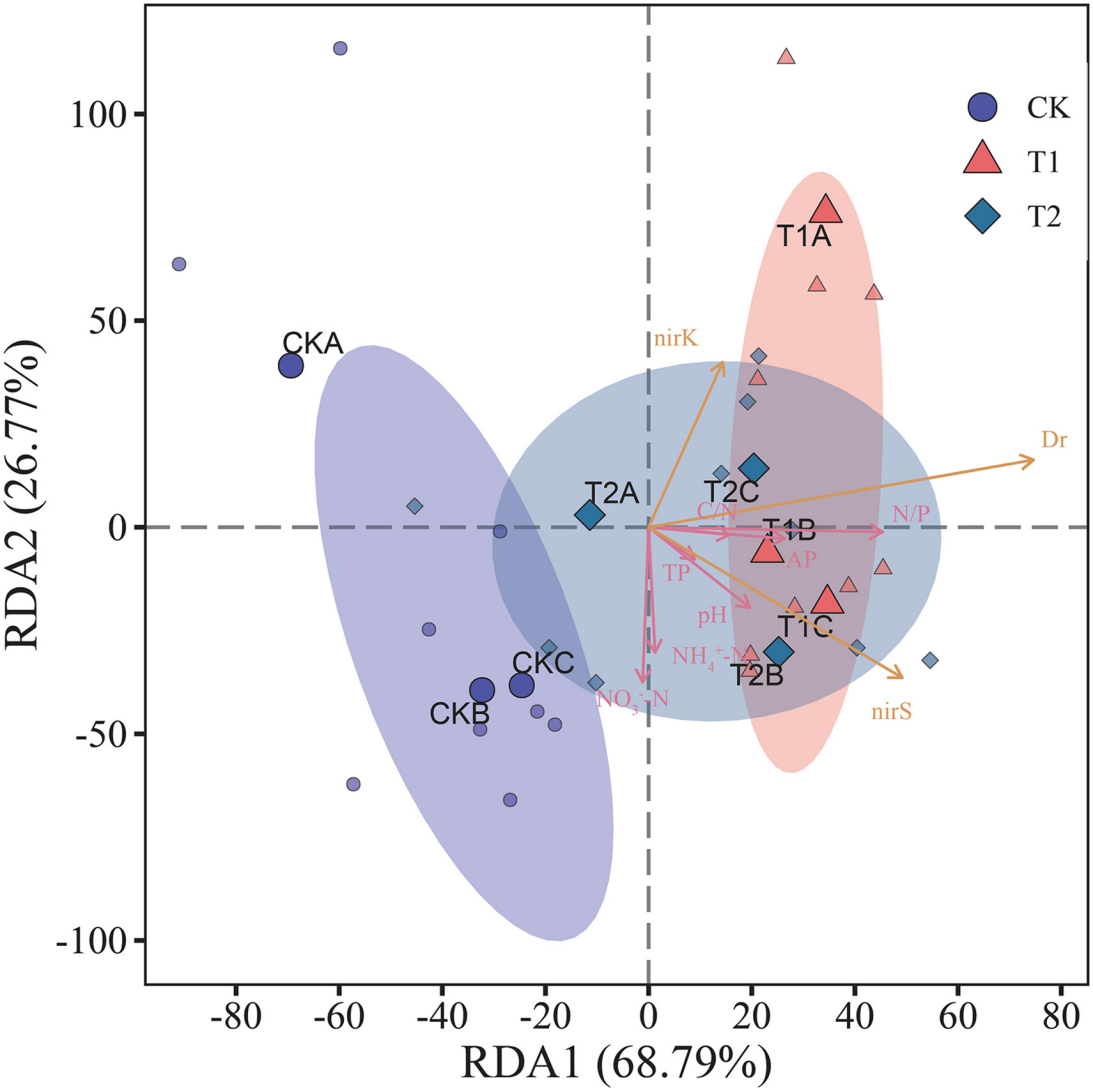
RDA ordination of denitrification-related responses constrained by soil physicochemical properties in the blueberry rhizosphere. The response variables were nirS, nirK and Dr. RDA1 and RDA2 explain 68.79% and 26.77% of the constrained variation, respectively. Circles, triangles and diamonds indicate CK, T1 and T2, respectively, and semi-transparent ellipses show the main distribution range of each treatment group. Orange arrows represent response variables, and pink arrows represent soil explanatory variables. The global permutation test was significant (*F* = 2.819, *P* = 0.004, *n* = 27).

Random forest analysis (Figure 6) cross-validated the above results. For Dr, N/P (17.63), AP (16.11), TN (16.02), and OM (14.83) had the highest contributions, consistent with the RDA and network results. For *nirS*, TN (10.50), NH_4_^–^-N (9.91), N/P (9.03), and OM (8.19) were the main predictors. For *nirK*, all predictor variables had relatively low importance values, with pH being the highest at 5.57, consistent with the ANOVA result that *nirK* was insensitive to the experimental treatments. For *virD2*, pH emerged as the dominant predictor, with an importance substantially higher than that of all other factors.

**FIGURE 6:**
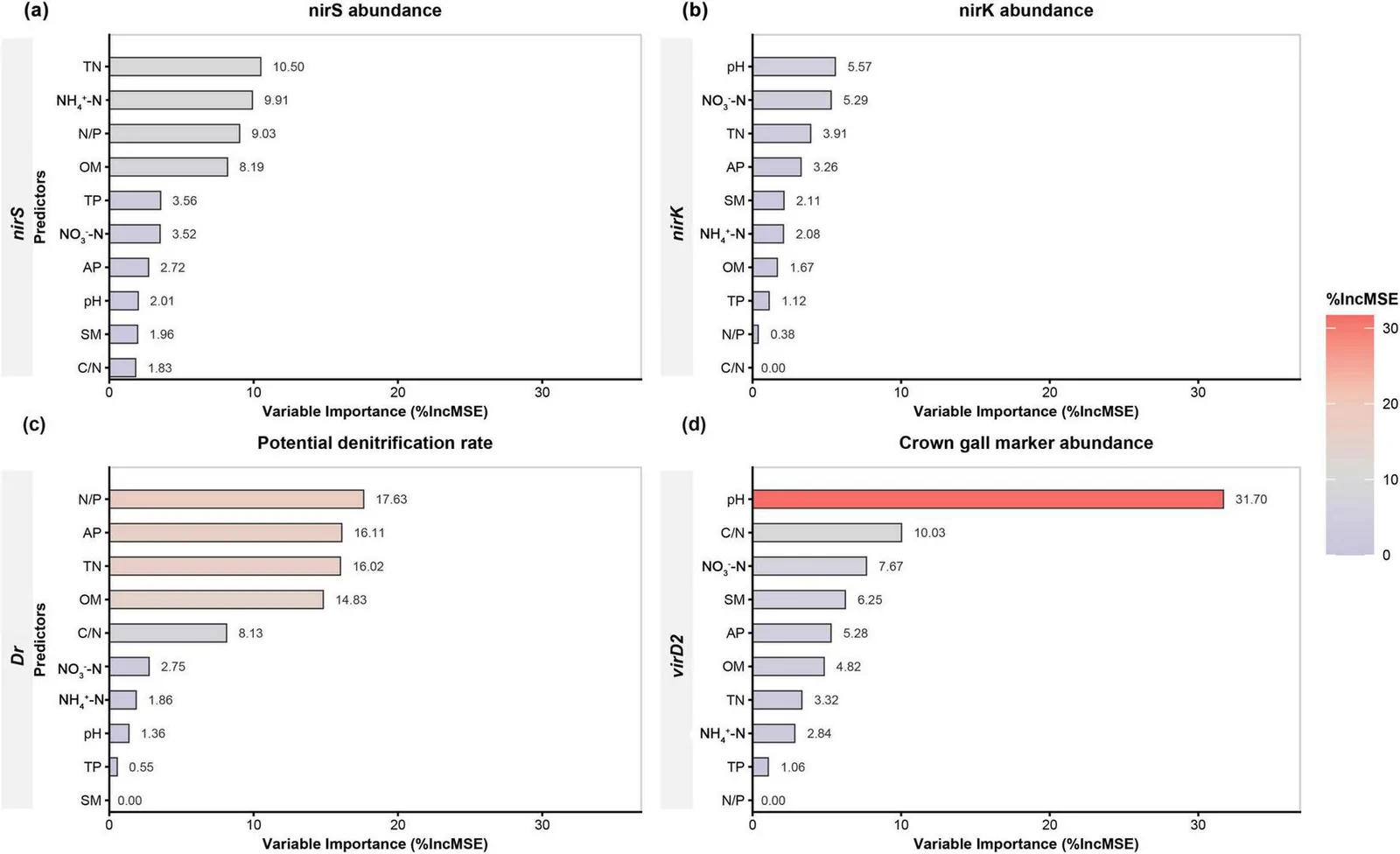
Random forest analysis identifies environmental predictors of nirS, nirK, potential denitrification rate and crown gall marker abundance. **(a)** *nirS* abundance. **(b**) *nirK* abundance. **(c)** Potential denitrification rate (Dr). **(d)** Crown gall marker abundance (*virD2*). Bars indicate mean variable importance across 200 repeated random forest regression runs, expressed as %IncMSE, and numbers at bar ends indicate the displayed importance values. Error bars are not shown. Bar colour represents the magnitude of variable importance. All models used soil environmental variables only as predictors.

As verified by the response fingerprint plot (Figure 7), TN, OM, AP, and Dr all showed positive responses under all six combinations of treatments and irrigation regimes. *nirS* and *nirK* also showed positive responses in most combinations, but the *nirS/nirK* ratio fluctuated between positive and negative values. *virD2* could be reliably estimated in only two combinations (T1 under high water and T1 under low water, corresponding to T1B and T1C in Figure 7), showing opposite directions (positive in T1B, negative in T1C).

**FIGURE 7:**
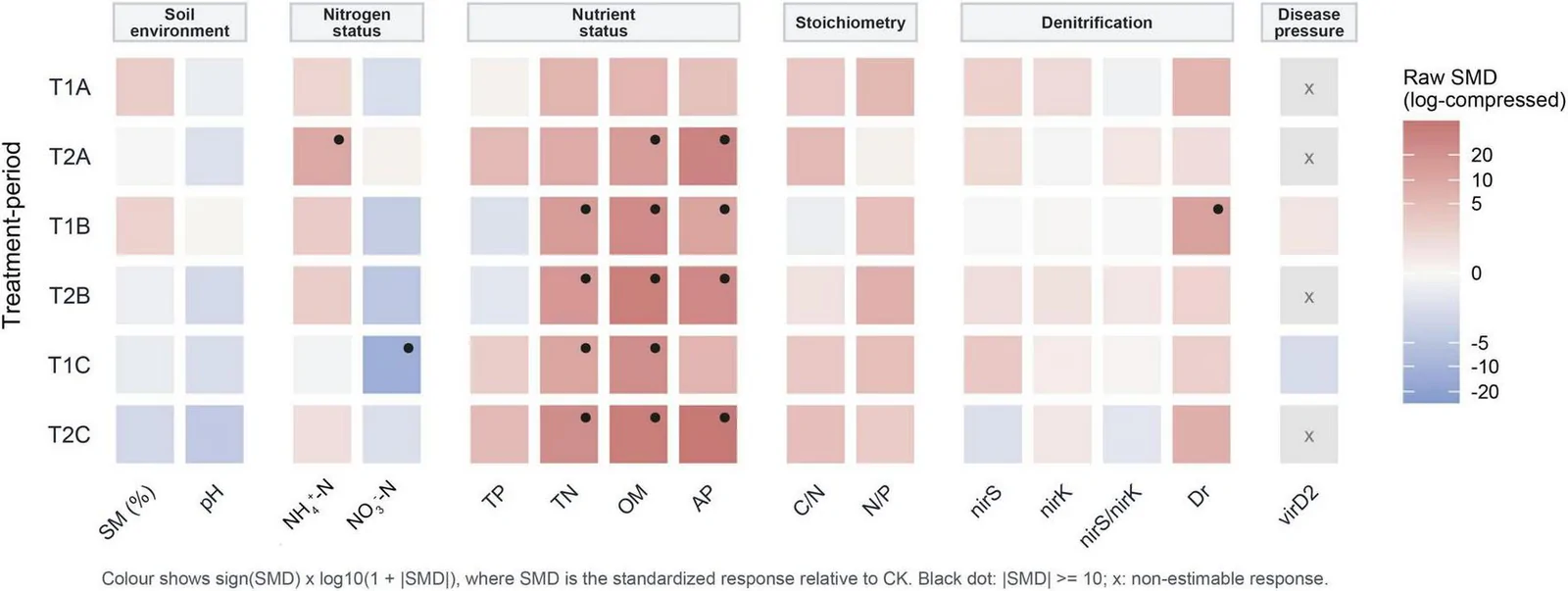
Standardized response fingerprint of soil amendment-induced changes across multiple indicators. The heatmap shows standardized responses of T1 and T2 relative to the corresponding CK within each water-period condition; CK is therefore not shown as a separate row. Colors represent sign (SMD) x log_10_(1 + | SMD|), with red indicating increases relative to CK, blue indicating decreases and near-white indicating weak responses. Black dot indicates extreme raw responses with | SMD| > = 10, and x indicates non-estimable responses (fewer than two quantifiable replicate abundances in a group, i.e., values at or below the reliable quantification limit recorded as zero in the log10-based SMD computation; see Methods).

### 3.5 Crown gall disease phenotypes

Crown gall symptoms were observed across all amendment–water regime combinations, indicating that amendments did not completely prevent infection under artificial inoculation (Table 1). However, the proportion of infected plants varied among treatments, disease incidence reached 100% in every treatment combination; in T2-W3 all three plants developed only grade-1 callus at the inoculation site, whereas CK and T1 had 100% incidence across all water regimes.

**TABLE 1:** Summary of blueberry crown gall disease incidence under different treatment regimens.

Treatment	Water regime	Infected plants (/3)	Incidence (%)	Disease index	Gall number	Max diameter (mm)	Fresh weight (g)
CK	W1	3	100	63.0 ± 12.8	3.33 ± 0.58	6.67 ± 1.61	0.627 ± 0.220
	W2	3	100	92.6 ± 12.8	5.00 ± 1.00	14.17 ± 2.84	1.640 ± 0.451
	W3	3	100	40.7 ± 12.8	2.33 ± 0.58	4.17 ± 0.91	0.317 ± 0.091
CK pooled		9	100	65.4 ± 24.4	3.56 ± 1.33	8.33 ± 4.74	0.861 ± 0.610
T1	W1	3	100	48.1 ± 12.8	2.33 ± 0.58	5.23 ± 1.14	0.423 ± 0.114
	W2	3	100	70.4 ± 12.8	3.67 ± 0.58	9.00 ± 1.50	0.817 ± 0.165
	W3	3	100	25.9 ± 12.8	1.33 ± 0.58	2.33 ± 1.17	0.153 ± 0.083
T1 pooled		9	100	48.1 ± 21.5	2.44 ± 1.13	5.52 ± 3.07	0.464 ± 0.301
T2	W1	3	100	18.5 ± 12.8	1.00 ± 0.00	1.50 ± 0.89	0.083 ± 0.059
	W2	3	100	40.7 ± 12.8	1.67 ± 0.58	4.43 ± 0.91	0.317 ± 0.091
	W3	3	100	11.1 ± 0.02	1.00 ± 0.01	0.83 ± 0.35	0.030 ± 0.01
T2 pooled		9	100	23.5 ± 13.9	1.22 ± 0.44	2.26 ± 1.79	0.143 ± 0.143

Disease index (DI) calculation formula:DI = [Σ(number of diseased plants in each grade × grade value) / (total plants × 9)] × 100.

Disease severity also differed substantially, with mean disease index decreasing from CK (65.4 ± 24.4) to T1 (48.1 ± 21.5) and T2 (23.5 ± 13.9). Two-way ANOVA showed significant effects of amendment and water regime on disease index (amendment: *F* = 27.38, *P* < 0.001, η^2^p = 0.75; water: F = 27.38, *P* < 0.001, η^2^p = 0.75), with no significant interaction (*F* = 0.75, *P* = 0.571, η^2^p = 0.14). Under W1, disease index declined from 63.0 (CK) to 48.1 (T1) and 18.5 (T2); corresponding values under W2 were 92.6, 70.4, and 40.7; under W3, 40.7, 25.9, and 3.7 (T2). T2 consistently produced the lowest disease severity across all three water regimes.

Gall growth patterns mirrored disease index. Mean gall number per plant decreased from 3.56 ± 1.33 (CK) to 2.44 ± 1.13 (T1) and 1.22 ± 0.44 (T2). Maximum gall diameter declined from 8.33 ± 4.74 mm (CK) to 5.52 ± 3.07 mm (T1) and 2.26 ± 1.79 mm (T2). Gall fresh weight decreased from 0.861 ± 0.610 g (CK) to 0.464 ± 0.301 g (T1) and 0.143 ± 0.143 g (T2).

Amendment and water regime had significant effects on gall number, maximum diameter, and fresh weight (all *P* < 0.001). The amendment × water interaction was not significant for gall number (*P* = 0.070), but was significant for maximum diameter (*P* = 0.012) and fresh weight (*P* = 0.002), indicating that water regime modulated the amendment effect on gall enlargement and biomass accumulation.

Water regime also influenced disease development. W2 yielded the highest disease index and greatest gall growth within each amendment, while W3 generally yielded the lowest. For instance, under CK, disease index rose from 63.0 (W1) to 92.6 (W2) then fell to 40.7 (W3), with similar patterns in T1 and T2. These results suggest that high water favored symptom development and gall enlargement post-inoculation, though this response was attenuated by amendment, especially T2.

Overall, while incidence was universal, amendments reduced disease index and restricted gall formation, enlargement, and biomass accumulation. The severity gradient CK > T1 > T2 aligned with *virD2* abundance patterns (CK > T1 > T2), but differed from Dr (highest in T1), indicating that crown gall severity reduction was more closely linked to pH-associated suppression of *virD2* abundance than to enhanced potential denitrification rate.

## 4 Discussion

### 4.1 Remodeling of the rhizosphere microenvironment by soil amendment and its potential impact on pathogens

Recent advances in blueberry crown gall management have focused on pathogen-side strategies, such as genomic diagnostic markers and bacteriophage biocontrol. However, their field application is often limited by environmental variability and strain specificity. An alternative and complementary approach is to modify the rhizosphere physicochemical environment to reduce pathogen colonization and virulence gene expression. Soil acidification via organic amendments offers a broad-spectrum strategy across Agrobacterium genotypes. In this study, we tested whether a mushroom-residue-based amendment could acidify the blueberry rhizosphere, reduce the abundance of the virulence marker *virD2*, and consequently decrease crown gall severity.

One-way ANOVA results for rhizosphere soil physicochemical properties showed that T2 consistently reduced soil pH across all water regimes, with the lowest value (4.99) observed under moderate water (W1), and exhibited greater acidification than T1. This difference is likely related to the addition of mushroom residue. One possibility is that organic matter within the residue provided carbon sources and attachment sites for sulfur-oxidizing bacteria, which may have indirectly facilitated microbial oxidation of elemental sulfur. Another possibility is that organic acids released during residue decomposition contributed to further pH reduction. Such synergistic effects between organic amendments and sulfur oxidation have been observed in previous studies (Phan and Sabaratnam, 2012; Lee S. I. et al., 2021). However, because we did not chemically characterize the mushroom residue (e.g., C/N ratio, pH, nutrient content, phenolic compounds, or microbial community), these interpretations remain speculative and require further verification. Apart from chemical changes, the physical effects of amendments on soil water-holding capacity were relatively limited, because water condition was always regarded as the primary factor determining soil water content throughout the experimental period; only under the water-deficit condition (W3) did the soil water content of the T2 treatment become slightly lower than that of the control (CK) and the T1 treatment, which may reflect either increased porosity after the addition of mushroom residue or enhanced hydrophobicity caused by organic matter (Wang A. et al., 2024). Although this physical effect did not translate into major treatment differences in SM, it may have indirectly influenced solute diffusion and oxygen availability in the rhizosphere under dry conditions.

Regarding nutrient patterns, total nitrogen (TN) and total phosphorus (TP) generally followed the order T2 > T1 > CK across all water regimes, with T2 showing particularly strong enrichment of TN. Peat and mushroom residue are inherently rich in organic matter and nutrients, and the higher microbial availability of mushroom residue favors nutrient immobilization (Chen et al., 2024). Studies on spent mushroom substrate have demonstrated that its application significantly increases soil total nitrogen, hydrolyzable nitrogen, and available phosphorus, and enhances rhizosphere microbial diversity (Yang et al., 2026), consistent with the present results. In most reported cases, the increase in total nitrogen following spent mushroom substrate application ranges from approximately 1.2- to 1.8- fold relative to unamended controls (Phan and Sabaratnam, 2012; Yang et al., 2026). The magnitude of TN enrichment observed in T2 (up to 2.6 times that of CK under low water) exceeds this general range, suggesting a possible synergistic effect between peat and mushroom residue that warrants further investigation. However, this comparison should be interpreted with caution, as soil background conditions and application rates vary considerably across studies. Notably, the treatment ranking of TP varied among water regimes, indicating that the phosphorus-supplying capacity of amendments was modulated by soil moisture. This interaction may reflect moisture-dependent changes in phosphorus solubility or microbial P-cycling activity, and suggests that the benefits of amendment for phosphorus nutrition are more sensitive to environmental moisture than those for nitrogen (Wang X. et al., 2024).

The C/N and N/P ratios also showed clear differentiation among treatments. T2 reached a C/N of 28.04 under moderate water (W1), approaching the threshold (approximately 30:1) above which high C/N stimulates microbial immobilization of inorganic nitrogen. This may explain why inorganic nitrogen (NH_4_^–^-N and NO_3_^–^-N) contents in T2 were not significantly higher than those in CK under low water (W3) (Cao et al., 2021). From a stoichiometric perspective, the elevated C/N ratio in T2 likely shifted the rhizosphere from net mineralization toward net immobilization, thereby reducing inorganic nitrogen availability despite the substantial total nitrogen pool. The N/P ratio, which varied significantly among amendment–water combinations, is increasingly recognized as a key stoichiometric constraint on microbial metabolism (Trautwein et al., 2017). The elevated N/P in T2 suggests that phosphorus availability may have limited microbial nitrogen utilization under certain moisture conditions, adding further complexity to the interpretation of inorganic nitrogen dynamics.

The combined effects of pH reduction and nutrient pattern alteration may indirectly influence rhizosphere pathogens. Previous studies have shown that pH is a primary environmental factor driving bacterial community structure in the blueberry rhizosphere (Che et al., 2022), and that low pH may exert selective pressure against Agrobacterium tumefaciens, which prefers neutral conditions. This pH-dependent suppression hypothesis is supported by previous findings that Agrobacterium tumefaciens vir gene induction is optimal at neutral pH and significantly reduced under acidic conditions (pH < 5.5) (Li et al., 2002; Binns and Zhao, 2020), and that crown gall incidence is generally lower in acidic soils than in calcareous ones (Yang et al., 2017). On this basis, this study speculated that the acidification effect generated by T2, in addition to altering the competitive balance of the native microbial community, might also directly interfere with the expression of pathogenicity genes. Accordingly, T2 could simultaneously regulate pH and nutrient molar ratios to construct a rhizosphere microenvironment that is unfavorable for pathogen colonization, although this mechanism still awaits experimental verification (e.g., through *in vitro* pH manipulation experiments and direct measurement of vir gene expression). From the perspective of sustainable management, maintaining rhizosphere pH within the range of 4.5–5.5 using amendments such as T2 represents a promising strategy for green crown gall disease control, which on the one hand reduces reliance on chemical fungicides and on the other hand aligns with the principles of integrated pest management (IPM). Meanwhile, the use of mushroom residue-an agricultural by product, to convert organic waste into a valuable soil amendment reflects the concept of a circular economy. Future research should focus on validating this pH based suppression mechanism under field conditions and exploring its compatibility with other biological control agents.

Collectively, the physicochemical responses described above confirm that water regimes primarily drove short-term inorganic nitrogen pulses, whereas amendment treatments exerted more persistent control over pH and total nutrient pools. This differentiation between short-term supply and long-term reservoir regulation has important implications for both plant nutrition and pathogen suppression, and provides the physicochemical context for interpreting the functional gene and disease phenotype responses examined in this study.

### 4.2 Differential responses of denitrifying communities and pathogen markers to amendment and water regimes

In this study, *nirS* abundance followed the order T2 > T1 > CK and was generally higher under high water (W2), whereas *nirK* showed no significant response to either amendment or water regime (*p* = 0.283). This gene-specific differentiation aligns with previous reports that *nirS*- and *nirK*-type denitrifiers differ in ecological niches and environmental sensitivities (Luo et al., 2020). Specifically, studies have shown that in high pH soils, the abundance of *nirS* exhibits a stronger correlation with denitrification activity, whereas in low pH soils, the abundance of *nirK* shows a stronger correlation with denitrification activity (Samad et al., 2016). Nevertheless, even under the strongly acidic condition (pH 4.99) of the T2 samples, *nirS* maintained its activity, suggesting that nutrient availability might have overridden the expected inhibitory effect of pH on the *nirS*-type community; this interpretation is corroborated by other findings, namely that soil pH and total nitrogen are the two dominant factors controlling the community composition of both *nirS* and *nirK*-type denitrifiers (Yang et al., 2017). The enrichment of *nirS* in T2, which also had the highest nutrient levels, supports a positive regulation of *nirS*-type communities by nutrient supply (Luo et al., 2020).

Dr followed the order T1 > T2 > CK and was also highest under high water, but exhibited greater within-group variation in T2 than in other treatments. Previous studies suggest that denitrification rates are often better predicted by instantaneous environmental factors (e.g., moisture and C/N availability) than by gene abundances alone (Tang et al., 2019; Binns and Zhao, 2020). Indeed, measurements of soil moisture, carbon, and nitrogen availability have been shown to be more useful predictors of denitrification rates than denitrifier abundance alone across landscape scales (Zhou et al., 2018), and soil moisture, soil organic carbon, and nitrate concentration predominantly regulate denitrification under different land use types (Chen et al., 2023). Several immediate environmental factors may explain why Dr was lower in T2 than in T1 despite higher *nirS* abundance. First, the C/N ratio in T2 reached 28.04 under moderate water (W1), approaching the threshold (approximately 30:1) above which microbial immobilization of inorganic nitrogen predominates over mineralization. This high C/N would reduce NO_3_^–^ availability, thereby limiting the substrate supply for denitrification. Second, the lower pH in T2 (4.99) may have suppressed the activity of denitrifying enzymes, as denitrification is generally more efficient at near-neutral pH. These two factors—substrate limitation and pH inhibition—likely offset the potential advantage of higher *nirS* abundance in T2, resulting in a lower measured Dr than in T1.

Additionally, the nitrate-residual method used to measure Dr assumes that all NO_3_^–^ consumption results from denitrification. In acidic, carbon-rich soils such as T2, DNRA may represent an alternative NO_3_^–^ consumption pathway, potentially leading to an underestimation of true denitrification rates in this treatment. This methodological constraint should be considered when interpreting the lower Dr values observed in T2.

The pathogenic marker gene *virD2* was detectable in all treatment groups, and its average abundance showed a progressive decline from the control (CK, 4.15 log10 copies⋅g^−1^) to T1 (3.35) and then to T2 (2.21), with the abundance under the W2 treatment being generally higher than that under W1 and W3. This abundance gradient was fairly consistent with the pH gradient among treatments (CK > T1 > T2). Notably, previous mechanistic studies have demonstrated that the vir gene induction system of A. tumefaciens is pH-sensitive: optimal vir gene expression occurs at near-neutral pH, and acidic conditions can reduce the transcriptional activation of key virulence regulators (Binns and Zhao, 2020). The pH value of the T2 rhizosphere (4.99) fell below this optimal induction threshold. However, because our qPCR measurement reflects *virD2* target-gene abundance rather than transcriptional activity or viable cell counts, we interpret the observed reduction in *virD2* copy numbers as an indicator of reduced pathogen abundance in the rhizosphere, rather than as direct evidence of suppressed vir gene expression. Nevertheless, the consistency between the pH gradient and the *virD2* abundance gradient is compatible with the hypothesis that acidic conditions may reduce pathogen colonization or persistence in the rhizosphere. Thus, rather than introducing a completely novel mechanism, our findings demonstrate that the T2 amendment effectively pushes the rhizosphere pH into a range already known to suppress Agrobacterium virulence gene expression. Specifically, the vir gene induction system of A. tumefaciens is sensitive to acidic pH, with its pathogenic genes achieving optimal induction at pH 5.5–5.7, and the *VirG* transcriptional activator undergoes transcriptional induction in acidic media (Subramoni et al., 2014). The pH value (4.99) of the T2 treatment group was below the optimal induction threshold, a condition that would suppress vir gene expression and thereby also suppress pathogen proliferation, ultimately leading to the lowest abundance of *virD2* in this treatment group; this pH-dependent suppression mechanism is further corroborated by the observation that crown gall disease severity is higher in alkaline soils than in acidic soils (Faist et al., 2016). It is worth noting that no significant correlation was detected between *virD2* abundance and the abundances of Dr or *nirS*, indicating that variations in pathogen marker abundance are not associated with the dynamics of the denitrifying microbial community (Ma et al., 2026).

The distinctly different response patterns between denitrification parameters and virD2 offer two insights. First, the difference in response patterns between Dr (highest in T1) and virD2 (lowest in T2) indicates that, under the tested conditions, enhanced denitrification potential does not directly cause a reduction in pathogen marker abundance. Second, virD2 shows a positive correlation with pH rather than with nutrient or denitrification parameters, suggesting that pH is a key factor associated with the reduced abundance of pathogen-related markers in the T2 treatment group. However, because T2 differed from T1 in multiple respects (lower pH, higher nutrient enrichment, and presence of mushroom residue-derived organic matter), the relative contributions of these factors to disease suppression could not be separated in this experimental design. Based on these findings, soil amendments (especially the T2 formulation) can be regarded as a supplementary strategy for creating a rhizosphere environment unfavorable for pathogen colonization, although the underlying mechanism appears to be more attributable to physicochemical regulation than to microbial competition through the nitrogen cycle. However, because T2 differed from T1 in multiple respects, including lower pH, higher nutrient enrichment (TN, TP, OM), and the presence of mushroom residue-derived organic matter, the relative contributions of these factors to disease suppression could not be separated in this experimental design. Therefore, the observed attenuation of crown gall severity in T2 should not be attributed solely to pH suppression.

From a practical standpoint, the pH-associated suppression pattern observed here implies that acidifying amendments could be integrated with other management practices to reduce pathogen pressure. However, because our data are correlational, we cannot establish a direct causal link between pH reduction and *virD2* suppression. Independent experiments with controlled pH manipulation are needed to verify this proposed mechanism.

From a practical standpoint, the pH-associated suppression pattern observed here implies that acidifying amendments could be integrated with other management practices to reduce pathogen pressure. Consistent with the Methods, the *virD2* abundance quantified in this study was derived from rhizosphere soil DNA rather than from gall tissue. Therefore, *virD2* copy numbers should be interpreted as an indicator of pathogen abundance in the rhizosphere (i.e., shed or root-surface associated bacteria) rather than as a direct measure of within-tumor pathogen biomass. Future studies quantifying pathogen load directly from gall tissues would help validate the relationship between *virD2* copy number and disease severity.

### 4.3 Limited role of denitrification in directly suppressing crown gall disease

The correlations among denitrification function, nutrient availability, and pathogen markers provide correlative evidence for potential mechanisms linking soil amendment to disease suppression. Spearman correlation and network analyses (Figures 3, 4) showed that Dr was significantly positively correlated with TN, OM, AP, and N/P, and served as a hub node connecting these factors. This suggests that enhanced denitrification depends on the coordinated supply of carbon, nitrogen, and phosphorus (Zhang et al., 2026). Similar carbon-nitrogen-phosphorus co-regulation of denitrification has been reported in agricultural and forest soils (Samad et al., 2016; Luo et al., 2020).

The positive *nirS*-TN/N/P correlations and the negative nirK-SM correlation further support the ecological niche differentiation between the two denitrifying groups observed above. This pattern is consistent with previous reports that *nirS*-and *nirK*-type denitrifiers respond differently to environmental gradients (Kou et al., 2021), with *nirS*-type communities being more resource-driven (responsive to TN and N/P) and *nirK*-type communities being more moisture-sensitive (negatively responding to SM). However, the direction and strength of these niche differentiations are not universal across ecosystems; some studies have reported that *nirS*-type communities are more sensitive to pH or moisture gradients than *nirK*-type communities in certain habitats (Yang et al., 2017), suggesting that the ecological preferences of these two denitrifying groups are context-dependent and may shift with the prevailing environmental filters of each ecosystem.

Notably, the correlation analyses also revealed a clear divergence in the environmental drivers of denitrification function versus pathogen markers. While Dr and *nirS* were primarily associated with nutrient variables (TN, OM, AP, N/P), *virD2* showed a markedly different association pattern: it was positively correlated with pH and negatively with the C/N ratio, but showed no correlations with Dr or any nitrogen or phosphorus nutrient indices. In the network, *virD2* maintained only a single edge to pH. This indicates that the pathogen marker was primarily associated with soil acidity rather than with nutrient supply, consistent with reports that crown gall is more severe in calcareous than in acidic soils (Binns and Zhao, 2020). Conversely, some field surveys have reported no significant correlation between soil pH and crown gall incidence across a narrow pH range (Sabri et al., 2024), suggesting that the suppressive effect of acidification may be threshold-dependent and only becomes apparent when pH drops below a certain level, as occurred in T2 (pH 4.99) but not in T1 (pH ≈ 5.4). The negative correlation observed between *virD2* and the C/N ratio may further reflect an indirect effect whereby an elevated C/N ratio promotes microbial nitrogen fixation, thereby potentially reducing nitrogen availability for pathogen proliferation (Cao et al., 2021), However, given the confounding between C/N ratio and pH across treatment groups, caution is warranted in interpreting this relationship. The link between C/N stoichiometry and pathogen suppression has been corroborated in other pathosystems, where high C/N amendments reduced disease severity by shifting microbial community composition toward competitively advantaged saprophytes; whether this mechanism operates similarly in the blueberry rhizosphere, remains to be verified (Termorshuizen et al., 2006).

Overall, the correlation patterns shown in Figures 3, 4 did not reveal a direct negative correlation between denitrification potential and pathogen markers. In contrast, these results reveal two distinct response patterns: nutrient-related variables were primarily associated with denitrification potential, whereas pH was more closely associated with the pathogen marker. This pattern contrasts with recent findings in other crop systems, which reported that denitrification directly promotes disease suppression by depleting nitrate or producing antimicrobial intermediates (Ghani et al., 2022). This study did not find such direct associations, suggesting that the relative importance of denitrification in disease suppression may vary with soil type, amendment composition, and the biological characteristics of pathogen infection. Therefore, although denitrification may contribute to broader nitrogen cycling benefits in amended soils, its direct suppressive effect on crown gall disease appeared limited under our experimental conditions. Importantly, these inferences are derived from correlational and multivariate analyses; they do not constitute evidence of causal regulatory pathways. The hypothesized relationships, which particularly include the causal role of pH in suppressing *virD2* and the independence of the denitrification and disease-suppression responses, require experimental validation through targeted manipulation of pH and nitrogen supply in future studies.

### 4.4 Amendment drives community differentiation, with N/P nutrients and pH governing Dr and virD2 respectively

Multivariate analyses further disentangled the relative contributions of environmental factors to denitrification function and pathogen markers.

RDA separated the samples along the RDA1 axis into CK (negative) and T1/T2 (positive), indicating that amendment was the primary driver of response variable differentiation, with its effect outweighing that of water regime. This separation pattern is consistent with recent findings in blueberry orchard soils, where organic amendments exerted a stronger influence on rhizosphere microbial community structure than seasonal moisture variation (Li et al., 2024). However, the magnitude of amendment-driven differentiation observed here (RDA1 explaining 68.79% of constrained variance) exceeds that reported in most comparable studies (Legendre et al., 2011), This result can be attributed to the combined acidifying and nutrient-enriching effects of the T2 formulation, which simultaneously altered multiple environmental filters. The inconsistent projections of Dr, *nirS*, and *nirK* in the ordination plot confirmed their differential responses to environmental gradients. The full model was significant (*F* = 2.819, *P* = 0.004), with N/P, NO_3_^–^-N, and NH_4_^–^-N all showing significant independent effects (*P* < 0.05). Random forest cross-validated N/P, AP, TN, and OM as core predictors of Dr, consistent with multivariate constraints reported in other ecosystems where denitrification is primarily regulated by nitrogen forms and stoichiometric ratios (Zhu et al., 2013; Kou et al., 2021). Notably, the predictor ranking differed from that reported in some agricultural soils, where soil moisture and temperature often emerge as dominant controls (Schaufler et al., 2010), suggesting that the relative importance of stoichiometric versus physical regulators of denitrification may vary with soil type and amendment history.

The response fingerprint plots showed that TN, OM, AP, and Dr all exhibited positive responses across all six combinations of treatments and water regimes, indicating that the effects of amendments on nutrient enrichment and denitrification were robust under different moisture conditions. This result is consistent with previous findings that organic amendments can enhance denitrification potential across a range of soil water regimes, although the magnitude of response may vary with amendment type and water content (Samad et al., 2016; Peng et al., 2025). Conversely, some studies have reported that the denitrification response to organic amendments may reverse under extreme moisture, particularly waterlogging or severe drought (Cayuela et al., 2013). This reversal was absent in study, probably because the moisture gradient (30–100% θWHC) was narrow and remained within the range supporting denitrifier activity. In contrast, *virD2* responses were detected in only two combinations (T1B and T1C in Figure 7, representing high and low water, respectively), with opposite directions (positive in T1B, negative in T1C) and less consistency than denitrification parameters, consistent with pH dependent regulation (Section “4.1 Remodeling of the rhizosphere microenvironment by soil amendment and its potential impact on pathogens”), since the vir gene induction system of A. tumefaciens is optimally induced at pH 5.5–5.7 (Hildebrand et al., 2001; Rodriguez-Mena et al., 2022); The pH of T2 (4.99), falling below the optimal threshold, may suppress vir gene expression, whereas the pH of T1 (approximately 5.4) remained closer to the optimal induction range, explaining the marked difference in virD2 responses between T1B and T1C; minor environmental variations near the pH threshold may shift the balance between induction and suppression of vir gene expression (Hildebrand et al., 2001).

Collectively, the results reveal two distinct response patterns. Nutrient variables were primarily associated with denitrification potential, whereas pH correlated more closely with virD2 (Figures 3, 4). The consistency between RDA and random forest analyses, together with the leave-one-out and partial RDA sensitivity checks, strengthens confidence in these findings. Nevertheless, the relatively high explanatory proportions in RDA (particularly RDA1) should be interpreted with caution, as they may partly reflect the strong treatment-induced differentiation inherent in this controlled pot experiment. Future studies with larger sample sizes and field validation are needed to confirm the generalizability of these patterns.

The consistency between RDA and random forest analyses strengthens confidence in these findings. The variable importance rankings from random forest (Figure 6) closely matched the predictor contributions identified by RDA (Figure 5), with N/P, TN, OM, and AP consistently emerging as core predictors for Dr, and pH for virD2. Although Figure 6 does not display error bars, the importance values were derived from 200 repeated random forest regression runs, and the high concordance between these two independent analytical approaches supports the robustness of the ranking. Nevertheless, we acknowledge that the relatively small sample size (*n* = 27) may limit the stability of the random forest model with multiple predictors; future studies with larger datasets are needed to further validate these findings.

Given the observational nature of our data and the exploratory use of multiple multivariate methods, the variable importance rankings and predictor associations reported here should be interpreted as correlational rather than causal.

### 4.5 Attenuation of crown gall severity by soil amendment under contrasting water regimes

An important finding of the disease assessment was that crown gall symptoms were observed across all amendment–water regime combinations, although the proportion of infected plants varied among treatments. While CK and T1 exhibited 100% incidence across all water regimes, T2 pooled incidence was disease incidence reached 100% in all treatment combinations, with only one infected plant in the T2-W3 combination (Table 1). Importantly, even in treatments where infection occurred, disease severity and gall growth were substantially reduced by amendment. The mean disease index decreased from CK (65.4 ± 24.4) to T1 (48.1 ± 21.5) and T2 (23.5 ± 13.9). Gall number, maximum gall diameter, and gall fresh weight showed similar decreasing trends, with T2 consistently producing the lowest values. These findings indicate that the amendments did not completely prevent infection under the artificial inoculation conditions, but substantially restricted subsequent symptom development and gall enlargement. Therefore, the effect of T2 should be described as attenuation of crown gall severity rather than complete prevention of infection (Hildebrand et al., 2001).

Disease development varied with water regime; disease index and gall growth were generally highest under W2 and lowest under W3 across all amendments, and water regime significantly affected disease index, gall number, maximum diameter, and fresh weight, indicating that water availability contributed to crown gall development after inoculation (Rodriguez-Mena et al., 2022). The significant amendment × water interaction on gall diameter and fresh weight indicated that water conditions modified the magnitude of amendment effects on gall enlargement and biomass accumulation. The practical implication of this interaction is that the reduction of gall size and biomass by T2 was most pronounced under W3 and weakest under W2, suggesting that water management could complement the T2 amendment strategy to maximize disease attenuation. However, since pathogen activity, plant physiological status, and soil oxygen availability were not measured separately, the observed water effect should not be interpreted as direct evidence of a specific moisture-mediated mechanism (Ahmad et al., 2025). Future studies integrating oxygen micro-sensor measurements and pathogen transcriptional activity assays would help resolve whether the water effect operates through host physiology, pathogen behavior, or altered rhizosphere oxygen status.

The T2 amendment (peat + mushroom residue + sulfur) recycles mushroom residue as a soil conditioner and suppresses pathogen markers via pH mediated effects. Its attenuation effect, observed across three water regimes (though weaker under high moisture), supports integrating T2 amendment with water management as a green strategy for crown gall control in cold region blueberry cultivation. Field scale validation is needed to confirm the long term efficacy and economic feasibility of this approach under diverse orchard conditions. It should be noted that the artificial needle-prick inoculation method used in this study bypassed natural physical barriers of the stem base, creating artificially susceptible infection sites. This likely contributed to the 100% infection incidence observed in CK and T1, which would be substantially higher than typical infection rates under natural field conditions. This method was chosen to provide a standardized and stringent challenge for evaluating amendment effects under high pathogen pressure, rather than to simulate natural infection processes. Therefore, the disease attenuation effects observed in this study should be interpreted as relative reductions in symptom severity under severe inoculation conditions, and field validation is needed to assess the practical applicability of these amendments under natural disease pressure.

### 4.6 Limitations

Several methodological limitations should be acknowledged. First, the one-way ANOVA used for gene abundances and Dr, rather than a full two-way ANOVA, was adopted to preserve statistical power under the limited sample size (*n* = 3). While this approach allowed us to detect overall treatment differences, it did not formally partition the variance contributed by amendment, water regime, and their interaction for these variables.

Second, the small sample size (*n* = 3 per treatment combination) may have limited the detection of subtle effects and increased the uncertainty of variance estimates, as reflected in the relatively large standard deviations for some disease indices (e.g., CK disease index: 65.4 ± 24.4).

Third, the experimental design did not include separate treatments for mushroom residue or sulfur alone. Consequently, the effects of pH reduction, nutrient enrichment (TN, TP, OM), and possible bioactive compounds in mushroom residue on disease suppression were confounded. The causal roles of individual amendment components require further investigation through factorial experiments with isolated factors.

Fourth, this study did not directly measure soil oxygen concentration, nitrate flux, or denitrifying enzyme activity, which limits our ability to fully resolve the drivers of the observed Dr patterns. Direct measurements of these factors in future studies would help clarify the mechanisms underlying the decoupling between *nirS* abundance and Dr.

Fifth, the random forest analysis included more than 10 predictors with only 27 samples, which may compromise the stability of variable importance rankings. The consistency between random forest and RDA results partially mitigates this concern, but future studies with larger sample sizes would strengthen the reliability of predictor identification. Similarly, the simultaneous use of multiple multivariate methods (RDA, random forest, stepwise regression, and correlation analysis) may increase the risk of overfitting; therefore, these results should be regarded as exploratory and hypothesis-generating.

Sixth, plant growth performance characteristics, such as shoot and root biomass, plant height, and photosynthetic activity, were not measured in this study. As a result, we cannot distinguish whether T2 reduced crown gall severity through direct pathogen inhibition or through enhanced host tolerance or resistance. Future studies integrating plant physiological measurements with rhizosphere analyses would help disentangle these potential mechanisms.

Seventh, the mushroom residue used in T2 was not chemically characterized for parameters such as C/N ratio, pH, nutrient content, phenolic compounds, or microbial community composition. This lack of characterization limits our ability to attribute the observed differences between T1 and T2 to specific properties of the residue. Future studies should include comprehensive characterization of amendment materials to enable more mechanistic interpretations.

Eighth, the artificial needle-prick inoculation method used in this study created wounds that circumvented natural physical barriers, likely overestimating infection rates compared to field conditions. The 100% incidence in all treatments reflects this artificially high pathogen pressure. Therefore, our findings on disease attenuation should be extrapolated to field conditions with caution, as natural infection processes may involve additional barriers and lower pathogen loads.

Ninth, the experimental design lacked a needle-wounding-only, non-inoculated control and did not include re-isolation and identification of A. tumefaciens from gall tissues. These omissions limit our ability to formally fulfill Koch’s postulates and to definitively attribute the observed galls solely to the inoculated strain. However, the positive correlation between *virD2* abundance and gall severity across treatments, together with the use of previously validated *virD2*-specific primers (Haas et al., 1995) confirmed by melt curve analysis, supports the association between pathogen presence and disease phenotype. Nevertheless, our conclusions regarding disease suppression are correlational and should be interpreted with appropriate caution.

Tenth, disease incidence (binomial) and severity scores (ordinal) were analyzed using ANOVA rather than binomial generalized linear models or ordinal regression, which would be more statistically appropriate for these data types. This choice was made due to the small sample size (*n* = 3 per treatment combination), which precluded reliable fitting of more complex models. The raw incidence data are reported in Table 1 to ensure transparency, but the statistical results for disease phenotypes should be interpreted with caution, and future studies with larger sample sizes should employ generalized linear models or nonparametric methods for binomial and ordinal responses.

Eleventh, representative photographs of crown gall symptoms for each treatment were not systematically archived during the experiment. As a result, we are unable to provide visual documentation of the severity gradation across treatments. This limits the quality of phenotypic presentation, and future studies should ensure standardized photographic records with scale bars for all treatments.

## 5 Conclusion

This study investigated the effects of three soil amendment treatments (CK, T1, T2) under different water regimes on rhizosphere physicochemical properties, denitrification functional gene abundances (*nirS/nirK*), potential denitrification rate (Dr), and crown gall severity in ‘Northland’ blueberry, with particular focus on the environmental responses of the pathogenicity marker gene *virD2*. The main conclusions are as follows:

(1) Distinct responses of soil properties to amendment and water regimes. Soil moisture was primarily governed by water regimes, with limited regulation by amendment. T2 consistently reduced soil pH across all water regimes (minimum 4.99), confirming the acidifying effect of the peat + mushroom residue + sulfur formulation. Total nitrogen and total phosphorus generally followed T2 > T1 > CK, with TN under T2 reaching 2.6 times that of CK under low water, indicating strong nutrient retention. Inorganic nitrogen showed pulse dynamics under high water and declines under moderate and low water, suggesting that its short-term supply was primarily regulated by water-driven mineralization processes.

(2) Gene-specific responses of denitrification function. *nirS* abundance followed T2 > T1 > CK and was higher under high water, whereas nirK showed no significant response to either factor, indicating distinct ecological niche differentiation between the two denitrifying groups. Dr followed T1 > T2 > CK, a different ranking from *nirS*, suggesting that factors beyond nirS abundance contributed to the observed Dr variation, with Dr co-regulated by both gene abundance and immediate environmental conditions. *virD2* abundance decreased in the order CK (4.15 log10 copies⋅g^−1^) > T1 (3.35) > T2 (2.21), indicating that the T2 formulation was associated with reduced pathogen marker abundance.

(3) Divergent environmental drivers for denitrification versus pathogen markers. Multivariate analyses identified N/P, AP, TN, and OM as core predictors of Dr, whereas *virD2* was significantly correlated only with pH and showed no association with Dr or nutrient indices. This divergence indicates that the environmental factors driving denitrification potential are largely distinct from those associated with the pathogen marker, with pH playing a unique and isolated role in the latter.

(4) Attenuation rather than prevention of crown gall. Crown gall symptoms were observed across all amendment–water regime combinations, although the proportion of infected plants varied: CK and T1 exhibited 100% incidence, whereas T2 pooled incidence was 100% incidence in all treatment combinations, with only grade-1 callus in T2-W3. Disease index, gall number, maximum diameter, and fresh weight decreased progressively from CK to T1 and T2. The disease severity pattern aligned with *virD2* abundance (CK > T1 > T2) but diverged from Dr (highest in T1), indicating that T2 attenuated symptom development and gall growth rather than completely preventing infection. The reduction in disease severity was more closely linked to pH-related reduction in *virD2* abundance than to enhanced denitrification potential. This interpretation is based on statistical associations; causal relationships remain to be verified through targeted experiments.

(5) Implications for sustainable blueberry production. The T2 amendment formula (peat + mushroom residue + sulfur) offers a dual benefit: it recycles agricultural waste (mushroom residue) while reducing pathogen-related markers through pH-associated suppression. However, its efficacy in reducing gall size was attenuated under high water, suggesting that integration with moisture management, particularly drainage during rainy periods, is essential to maximize disease attenuation.

Future research should focus on field-scale validation of this pH-associated amendment strategy across diverse orchard conditions and its integration with compatible biocontrol agents for the sustainable management of blueberry crown gall. Although the observed associations between T2 amendment, reduced pH, decreased *virD2* abundance, and attenuated gall severity are consistent with the hypothesis of pH-related disease suppression, our experimental design does not establish causation. These findings should be validated in future studies with appropriate controls (e.g., non-inoculated treatments, pH-manipulated systems) and direct pathogen re-isolation.

## Data Availability

The raw data supporting the conclusions of this article will be made available by the authors, without undue reservation.
